# A Case Report of Rickets Due to Severe Nutritional Deficiencies

**DOI:** 10.7759/cureus.30095

**Published:** 2022-10-09

**Authors:** Arwa Saber, Nada Naaman, Rahaf Alqurashi, Rawia F Albar

**Affiliations:** 1 Medicine and Surgery, King Saud Bin Abdulaziz University for Health Sciences College of Medicine, Jeddah, SAU; 2 Pediatrics, King Saud Bin Abdulaziz University for Health Sciences College of Medicine, Jeddah, SAU

**Keywords:** anemia, malnutrition, vitamin d deficiency, delayed development, nutritional deficiencies

## Abstract

A 19-month-old boy presented to the general pediatric clinic with delayed development and multiple nutritional deficiencies, after being exclusively breastfed up to the age of nine months without vitamin D supplementation. Upon examination, imaging studies, and lab tests, the patient was diagnosed with nutritional rickets. The management included supplementation of cholecalciferol, ferrous sulfate, calcium carbonate, and multivitamin drops to support his diet, and was encouraged to follow a healthy balanced diet. Upon follow-up at the age of 20 months, the patient showed slight improvement and was able to walk, while at 22 months, the patient was developmentally up to age, and had a good appetite with a slight increase in weight. Despite the high incidence of nutritional deficiencies, there is still a lack of awareness and late presentations of such cases, which can lead to complications if not detected early. This case demonstrates the importance of prevention of similar cases by early education about adequate nutrition to the patients and caregivers and regular follow-ups with the general practitioner for early detection and early supplementation as required.

## Introduction

Vitamin D is a lipid-soluble vitamin that is mainly produced by the body when ultraviolet rays from sunlight make contact with the skin and activate its synthesis, and can also be found in some foods and supplements. It plays a major role in the intestinal absorption of calcium, magnesium, and phosphate, which promotes bone mineralization and growth [1]. Previous studies have also shown that vitamin D can reduce cancer cell growth, help control infections and reduce inflammation [1].

Vitamin D deficiency is a highly common epidemic and health problem affecting 86.27% of children living in Jeddah, Saudi Arabia [2]. People who are dark-skinned, obese, on a vegan diet, or have limited exposure to sunlight are at a higher risk for developing vitamin D deficiency [3]. Research has shown that vitamin D deficiency has serious clinical consequences, including cardiovascular diseases, cancer, and diabetes. It can also lead to loss of bone density, which contributes to osteoporosis and fractures [4]. 

In children, severe vitamin D deficiency can lead to rickets. Rickets is a common childhood skeletal disorder that causes the bones to soften and become weak resulting in bone deformities [5]. The patients can present with symptoms of bone pain, poor growth, and delayed motor skills [5]. This includes fatigue, bone pain, depression, hair loss, muscle weakness, and loss of appetite. Complications of rickets include failure to thrive, abnormally curved spine, bone deformities, dental defects, and seizures.

In this case report, we describe a 19-month-old infant as a case of nutritional rickets caused by severe nutritional deficiencies including vitamin D, zinc, ferritin, magnesium, and sodium.

## Case presentation

A 19-month-old male child was referred to the general paediatric department by his family physician as a highly suspected case of severe rickets. The child was born at term by an emergency caesarean due to multiple previous caesarean deliveries with a birth weight of 3.23 kg and length of 51 cm, within the 90th centile range. He cried actively at delivery with an Apgar score of 9/10 at one and five minutes and suffered no complications. He was exclusively breastfed up to nine months of age, without any vitamin D supplementation. His vaccinations were up to date and he had no known allergies. He had four older siblings and no family history of a similar condition. Upon the first presentation at the age of 17 months, to the family physician, the child’s weight was 7.97 kg, his height was 70 cm, and his head circumference was 47 cm, which all fall below the fifth centile.

**Figure 1:**
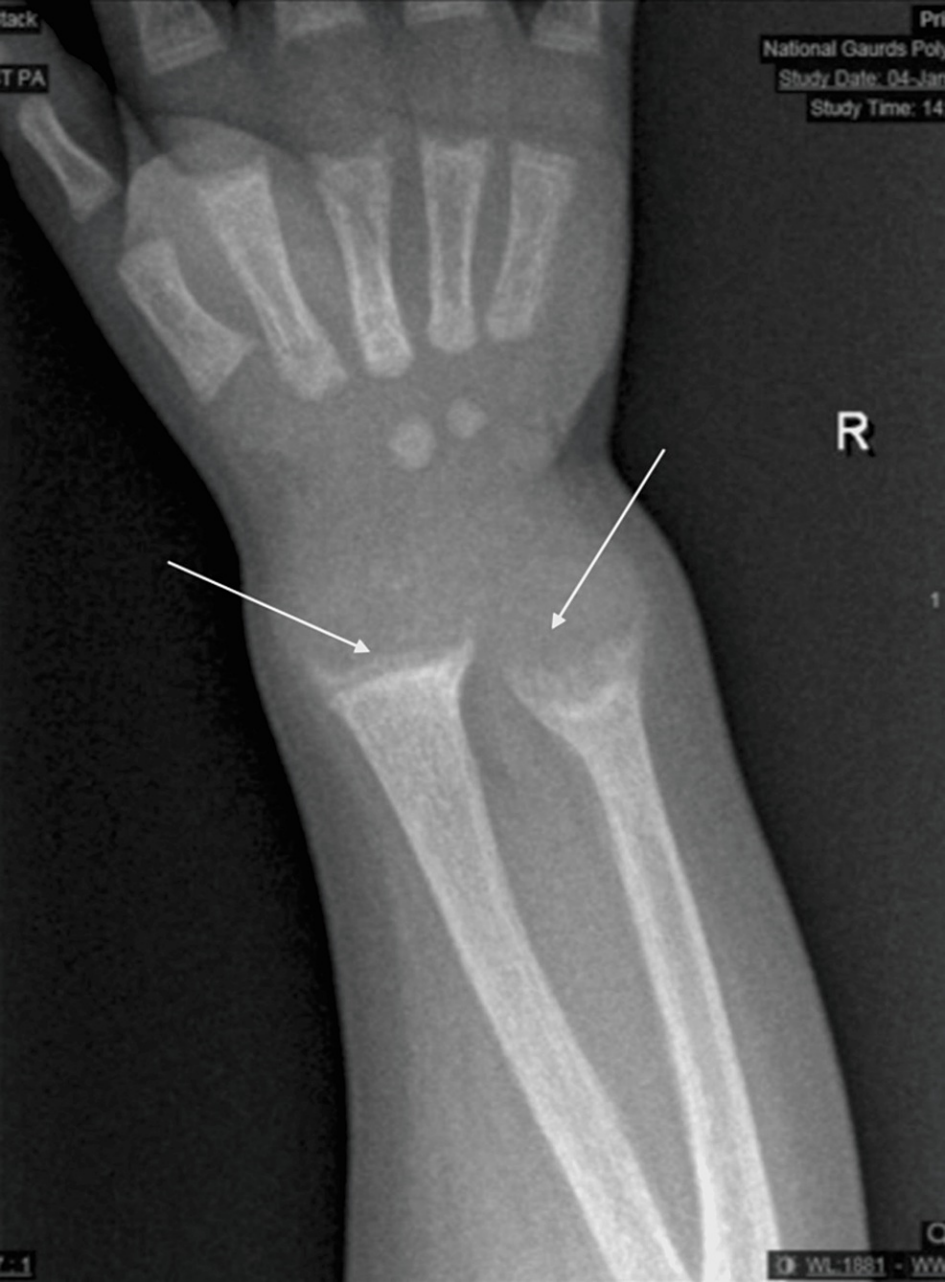
X-ray of the wrist showing fraying, widening, and cupping of the metaphyseal ends of both the ulna and radius bones.

On examination, it was seen that developmentally, he was delayed and could not walk unsupported. It was observed that there was marked widening of the wrists and bowing of the legs. Harrison sulcus was noted, but the rachitic rosary was not visible. Apart from an umbilical hernia, the remaining examination, including cardiothoracic and neuromuscular examinations, revealed no additional findings. Initial blood tests revealed elevated alkaline phosphatase (>2200 U/L; normal range (n): 156-369) and low hypophosphatemia (0.64 mmol/L; n: 1.38-2.19), adjusted calcium (2.19 mmol/L; n: 2.25-2.75), and 25-hydroxyvitamin D levels (19.3 nmol/L; n: 50-250); albumin levels were slightly above the normal range (49 g/L; n: 38-47). Wrist X-ray findings were consistent with the clinical findings showing fraying, widening, and cupping of the metaphyseal ends of both the ulna and the radius. Defects in mineralization were evident yielding a picture of active rickets with rachitic changes (Figure 1).

The patient was started on treatment for his multiple nutritional deficiencies including vitamin D and calcium rickets at the age of 19 months. He had no basis for hereditary rickets or other risk factors apart from the diet; being exclusively breastfed till the age of month months, and was then on a regular family diet including fruits, vegetables and meat. He was supplemented with cholecalciferol oral solution 400IU and then 2000IU drops for six weeks.

At his follow-up at 20 months of age, a low haemoglobin level (6.7 g/dL; n: 11.0-14.7) was detected. The child had no history of pallor, chest pain, shortness of breath, fatigue, or bleeding. His parents had never noticed signs of jaundice, change in urine or stool colour, or change in bowel habits. He had no family history of thalassemia, sickle cell anaemia, or glucose-6-phosphate dehydrogenase deficiency although the mother had a history of iron deficiency anaemia prior to her pregnancy. After further testing of ferritin levels, the patient was diagnosed with microcytic hypochromic iron deficiency anaemia. Follow-up lab tests showed that the patient had secondary hyperparathyroidism: hypophosphatemia (0.64 mmol/L; n: 1.38 - 2.19), adjusted calcium (2.35 mmol/L; n: 2.25 - 2.75), low 25-hydroxyvitamin D levels (19.3 nmol/L; n: 50 - 250) and high parathyroid hormone (170 pg/mL; n: 14 - 65 pg/mL), which can be another factor that may contribute to anaemia in rickets [6].

Following the lab results at 20 months, he was started on ferrous sulfate 6mg/kg/day for his microcytic hypochromic anaemia for months. In addition, he was also started on calcium carbonate 65mg/kg/day for two weeks. Multivitamin drops 5ml orally was prescribed to support his diet. Cholecalciferol oral solution was increased to 4000IU for four months. Evaluating his socio-motor skills, at 20 months, he showed slight improvement and was able to walk forward but not backward, and he could not run. He was also referred to Endocrinology to follow up on his failure to thrive.

At the latest follow-up at 22 months, he was able to kick a ball, use a spoon and fork, and use six words. He was developmentally up to age, could walk, run, go up and down the stairs (with two feet on the same step), say "baba" and "mama", and other words with two-word sentences, follow simple commands, and express his needs. The patient showed more improvement regarding the symptoms of rickets with a good appetite and a slight increase in weight.

The patient had also been referred to a dietician for diet guidance and at his latest follow-up at 22 months, he was on a regular family diet, which includes two meals (rice, meat, chicken) and was still breastfed six to seven times daily according to the child's needs. The patient was booked for regular check-ups every two months.

## Discussion

This report presents a case of severe multiple nutritional deficiencies including hypophosphatemic rickets that was due to being exclusively breastfed and the absence of vitamin D supplementation throughout the first nine months of life. Unfortunately, by the time the patient sought medical assistance, he had already become symptomatic and was developmentally delayed. Rickets is an example of severe vitamin D deficiency, with a peak incidence between three and 18 months of age [7]. This case could have been easily prevented by a systematic supplementation from birth to the age of 12-18 months. The European Society for Paediatric Gastroenterology, Hepatology and Nutrition recommend vitamin D as a supplement for breastfed infants, which should start during the first months of life and continue throughout childhood and adolescence to prevent deficiencies [7]. The optimal daily intake of vitamin D for these groups is 400IU daily [8]. In addition to vitamin D deficiency, the quantity and quality of calcium consumption is important and are highly dependent on the maternal diet, especially in cases of breastfed infants [8]. Therefore, calcium supplements are also recommended in exclusively or partially breastfed infants as it highly depends on the maternal diet such as the quality of the vegetarian or vegan mother’s supplementation, which can lead to nutritional deficiencies. It is, therefore, necessary to provide parents with awareness and regular checkups every two to six months in order to avoid potentially irreversible consequences.

A previous case was reported with nutritional rickets due to a lack of vitamin D supplements after birth and presented with clinical signs of rickets, suggesting hypocalcaemia as the cause of afebrile seizures [9]. The baby was successfully managed following the intravenous administration of calcium gluconate, oral supplementation with calcium and vitamin D, and dietary changes. The patient was also given iron supplements, as iron-deficiency anaemia was also detected. 

Iron deficiency anaemia has been associated with exclusive breastfeeding as well as rickets and anaemia. Consequently, in this case, exclusive breastfeeding could be said to have led to rickets and anaemia. Vitamin D deficiency has also been associated with a greater risk of anaemia, lower mean haemoglobin, and higher usage of erythrocyte-stimulating agents [10]. 

## Conclusions

Multiple nutritional deficiencies including Vitamin D, iron, and zinc deficiency are very common, especially in exclusively breastfed infants and can be prevented by early education, regular follow-ups every two to six months for early detection and early supplementation. Therefore, this case report adds to the literature about the importance of raising awareness of nutritional deficiencies due to exclusive breastfeeding after a certain age, adding solid food and supplements to the diet, and early treatment.
